# The Impact of Earthquakes on Public Health: A Narrative Review of Infectious Diseases in the Post-Disaster Period Aiming to Disaster Risk Reduction

**DOI:** 10.3390/microorganisms11020419

**Published:** 2023-02-07

**Authors:** Maria Mavrouli, Spyridon Mavroulis, Efthymios Lekkas, Athanassios Tsakris

**Affiliations:** 1 Department of Microbiology, Medical School, National and Kapodistrian University of Athens, 11527 Athens, Greece; 2 Department of Dynamic Tectonic Applied Geology, Faculty of Geology and Geoenvironment, School of Sciences, National and Kapodistrian University of Athens, 15784 Athens, Greece

**Keywords:** infectious diseases, earthquakes, landslides, gastrointestinal, respiratory infection, water-borne disease, vector-borne disease, wound infection, skin infection, disaster risk reduction

## Abstract

Earthquakes are among the most impressive natural phenomena with very high potential to set off a chain of effects that significantly affects public health through casualties and injuries. Related disasters are attributed not only to the strong ground motion and coseismic phenomena but also to secondary effects, comprising mainly landslides and tsunamis, among others. All these can create harsh conditions favorable for the emergence of infectious diseases that are capable of causing additional human and economic losses and disruption of the emergency and recovery process. The present study comprises an extensive narrative review of the existing literature on the earthquake-triggered infectious diseases recorded worldwide, along with their symptoms, causative pathogens, associated risk factors, most vulnerable population groups, and prevention strategies. Respiratory, gastrointestinal, and vector-borne diseases, as well as wound and skin infections, are mainly recorded among the earthquake-affected population. Measures for effectively preventing earthquake-triggered infectious diseases are also proposed. One of the widely proposed measures is the establishment of a proper disease surveillance system in order to immediately and effectively identify the pre- and post-disaster occurrence of infectious diseases. This approach significantly contributes to disease trends monitoring, validation of early warning, and support of the emergency response and recovery actions.

## 1. Introduction

Earthquakes are among the most impressive geological processes and remain one of the most unpredictable natural disasters, with the potential to cause destructive effects on humans and structures. Based on an overview of the last 20 years disasters, 552 earthquakes occurred, accounting for 8% of all disasters worldwide and ranking third after floods (3254 events, 44% of the total) and storms (2043 events, 28% of the total) [1].

Despite the low percentage of earthquakes in the total number of disasters worldwide from 2000 to 2019, and despite the quantitative predominance of mainly hydro-meteorological phenomena, earthquakes are among the deadliest events that can also cause mega-disasters with tens to thousands of human casualties, injured, and homeless people in earthquake-affected areas. In terms of the economic impact, earthquakes have caused economic losses of 636 million over the above period (21% of the total), an amount that assigns earthquakes third place on the list after storms ($1.39 trillion, 47% of the total) and floods ($651 billion, 22% of the total) [1].

Several factors are responsible for the high mortality and morbidity of earthquakes. They can be classified into factors related to the seismotectonic setting and the geotechnical regime of the affected area, the time of earthquake occurrence, the weather conditions during the post-disaster period, the demographic characteristics of the affected area, the social, cultural, and community characteristics, and the structural characteristics of the built environment [2,3,4,5,6,7,8,9,10,11,12].

Earthquakes cause a multitude of accompanying and subsequent phenomena [13,14] with considerable impact on the natural and built environment and consequently on the people who live and operate within them.

Earthquake casualties can be classified as instantaneous, rapid, or delayed [15,16,17,18]. Instantaneous casualties are caused by severe crushing skull and chest injuries resulting in external or internal hemorrhage or by drowning by earthquake-induced tsunamis. Rapid casualties occur within minutes or hours and are attributed to asphyxia from dust inhalation or chest compression, hypovolemic shock, or exposure to harsh environmental conditions. Delayed casualties occur within days and are attributed to dehydration, hypothermia, hyperthermia, crush syndrome, wound infections, or postoperative sepsis.

Τhe main causes of earthquake casualties worldwide comprise (i) the collapse of buildings due to ground shaking [6,19,20,21,22,23,24,25,26,27,28,29,30,31,32,33,34,35,36,37,38,39,40,41] (ii) drowning and burying by tsunami deposits left onshore or offshore and entrapment inside collapsing buildings and severe injuries during the run-up and the backwash phase of tsunami [42,43,44,45], (iii) the generation of post-earthquake fires [46,47,48], and (iv) the generation of earthquake-induced slope movements [49,50,51,52].

Major injuries are mainly attributed to the partial or total collapse of buildings, movement of landslide material, generation of post-earthquake fires, physical impact with debris in fast-flowing tsunami water, and spill of hazardous chemicals [12,53,54,55]. They vary from minor cuts and bruises to serious fractures, crush injuries and burns. They also include airway obstruction or asphyxiation from the large quantities of dust and debris generated by collapsing buildings [12].

As regards the earthquake effects on survivors’ mental health, the most common psychiatric conditions seen among earthquake survivors are post-traumatic stress disorder (PTSD) and major depression (MD) [56]. The prevalence of PTSD following the earthquakes has been reported to be between 1.20% and 87%, depending on various cultural and socio-demographic features [57,58,59,60,61,62,63].

From the abovementioned, it is clear that earthquakes have the potential to set off a chain of events that significantly affect public health and can also create adverse conditions in the affected communities, favorable for the occurrence of sporadic cases, outbreaks, and epidemics of infectious diseases.

Although it is impossible to accurately predict which diseases would be transmitted after a certain type of natural hazard, infectious diseases can be categorized as either water-borne, air-borne, or vector-borne diseases, or contamination from wounded injuries [64]. Infectious diseases may appear during the post-impact phase, which lasts for 4 days to 4 weeks. When a victim has contracted an infection with a long incubation period or a latent infection, the clinical manifestation of their symptoms may be identified during the recovery phase, 4 weeks after the disaster occurrence [64]. During this phase, both newly imported diseases and those that are already endemic in the disaster-affected area could spread and turn into epidemics.

The aim of this study is to conduct an extensive narrative review of the existing scientific literature to present the infectious diseases associated with earthquakes and their accompanying effects that occurred worldwide. Furthermore, their symptoms and their causative pathogens are also reviewed, along with the risk factors associated with the occurrence of infectious diseases. This review also highlights the magnitude of earthquakes followed by infectious diseases, as well as their distribution in active and seismic fault systems worldwide.

Based on the data of this review, as well as lessons learned and good practices from emergency responses to parallel occurrence and evolution of geophysical and biological hazards in recent years, the most effective prevention strategies to mitigate the adverse effects of earthquakes on public health will be highlighted and presented in a multi-hazard frame. The proposed measures aim at protecting all involved in an emergency situation, starting from the affected local population and the most vulnerable population groups up to the first responders, including Civil Protection staff, health workers, members of voluntary groups, and staff of emergency shelters.

## 2. Search Strategy

For this narrative review, all major medical, scientific, and technical research databases and resources contained in the National Center for Biotechnology Information (NCBI), part of the National Library of Medicine (NLM), were thoroughly investigated to identify documented sporadic cases, outbreaks, and epidemics of infectious diseases in humans around the world, which were considered to be associated with earthquakes and earthquake-triggered phenomena, including landslides and tsunami. More specifically, keyword searches were conducted on PubMed, Scopus and ScienceDirect.

The search terms were based on the World Health Organization (WHO) document “Communicable diseases following natural disasters: risk assessment and priority interventions” [65]. A list of known pathogenic microorganisms was compiled and used to generate key search terms to identify earthquake-associated infectious diseases, usually as a result of injuries, disruption of sanitation, movement of populations, and crowding. All published articles and official reports in English were searched with the specific search terms in the title, abstract, or keywords. To incorporate articles from scientific journals and official reports not included in the above-mentioned databases, an online search was conducted using relevant keyword phrases and related combinations using Google and GoogleScholar advanced searches.

No standard definition of what constitutes an infectious disease outbreak was used to avoid omitting potentially relevant studies of public health significance. Additionally, no filters were used to identify specific study designs.

## 3. Spatial Distribution and Parameters of Earthquakes Associated with Infectious Diseases during Post-Disaster Period

Sporadic cases, outbreaks, and epidemics of infectious diseases in humans induced by earthquakes and their accompanying phenomena, including landslides and tsunamis, were documented worldwide at various points in time, since the review was not focused on a specific time period. Based on the available related information, it was found that the earthquakes associated with the occurrence of infectious diseases fell within a time period from 1980 to 2016.

**Figure 1:**
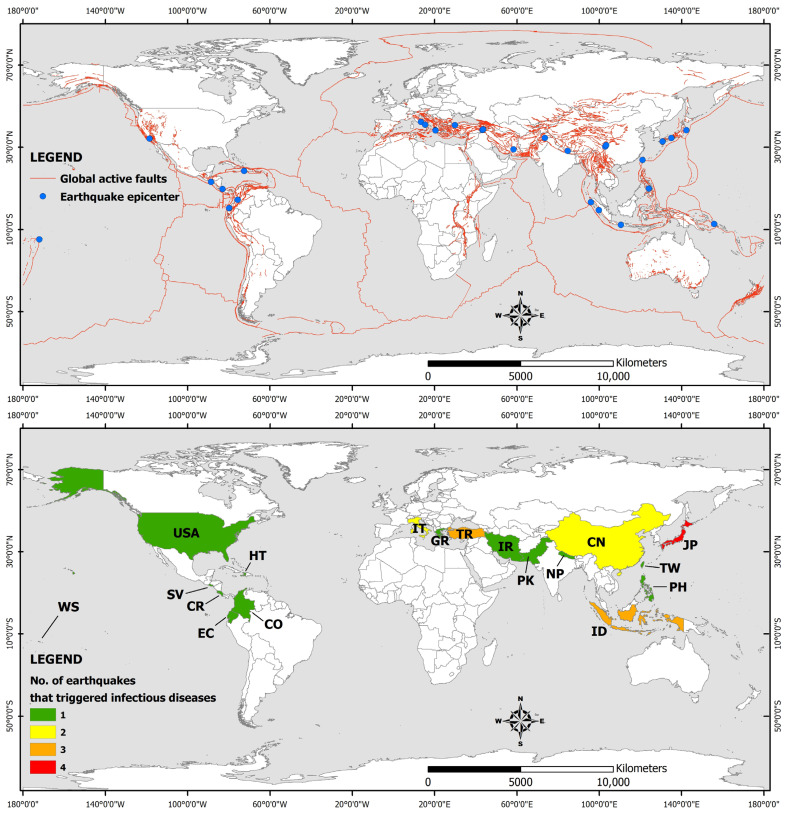
(**Up**) Map illustrating the active faults of the world and the epicenters of the earthquakes included in the study. (**Down**) Distribution of the countries affected by earthquake-triggered infectious diseases. The affected countries are located within the red fault zones. The global fault zones are from Styron and Pagani [67]. WS: Samoa, USA: United States, HT: Haiti, SV: El Salvador, CR: Costa Rica, EC: Ecuador, CO: Colombia, IT: Italy, GR: Greece, TR: Turkey, IR: Iran, PK: Pakistan, NP: Nepal, CN: China, ID: Indonesia, PH: Philippines, TW: Taiwan, JP: Japan.

The main parameters of the studied earthquakes such as the moment magnitude, the intensity and the number of human losses, injured people, and total affected people are summarized in Table 1. Their epicenters are presented in Figure 1, along with the countries affected by the earthquake-triggered infectious diseases.

**Table 1:** Parameters and impact of earthquakes generated within a time period spanning from 1980 to 2016 that induced infectious diseases and public health risks (Mw: moment magnitude, I: Intensity, HL: Human losses, IP: Injured people, TA: Total affected people). Information about Mw, I, HL, IP, and TA are extracted from the International Disaster Database EM-DAT [66].

No	Earthquake Occurrence (Date/Month/Year)	Epicentral Area	Affected Areas	Mw	I	HL	IP	TA
1	23/11/1980	Irpinia, (Italy)	Campania, Naples, Salerno	6.9	X	4689	7700	407,700
2	22/04/1991	Limon, (Costa Rica)	Costa Rica, Panama	7.7	X	47	199	10,419
3	17/01/1994	Northridge (California United States)	Greater Los Angeles area, Southern California	6.7	IX	60	7000	27,000
4	17/01/1995	Kobe (Japan)	Japan	6.9	XI	5297	34,492	541,636
5	25/01/1999	Armenia (Colombia)	Armenia, Pereira	6.2	X	1186	8563	1,205,933
6	17/08/1999	Izmit (Turkey)	Marmara area, Adapazari, Gölcük, Izmit and Yalova	7.6	X	17,127	43,953	1,358,953
7	21/09/1999	Chi-Chi (Taiwan)	central Taiwan Nantou County Taichung County	7.7	X	2264	8664	108,664
8	13/01/2001	El Salvador	El Salvador, Guatemala, Honduras	7.7	VIII	844	4723	1,334,529
9	26/12/2003	Bam (Iran)	Kerman Province	6.6	IX	26,796	22,628	267,628
10	26/12/2004	Indonesia	Indian Ocean coastal countries	9.2	IX	165,708	-	523,898
11	08/10/2005	Kashmir (Pakistan)	Pakistan, India	7.6	XI	86,000–87,351	69,000–75,266	2,800,000
12	27/05/2006	Yogyakarta (Indonesia)	Yogyakarta, Java, Indonesia	6.4	IX	5749–5778	38,568–137,883	600,000–699,295
13	02/04/2007	Solomon Islands	Solomon Islands, Papua, New Guinea	8.1	VIII	52	9	2384
14	12/05/2008	Sichuan (China)	Sichuan Province	7.9	ΧΙ	87,476	366,596	45,976,596
15	06/04/2009	L’Aquila (Italy)	Abruzzo	6.3	Χ	295	1000	56,000
16	30/09/2009	Sumatra (Indonesia)	Sumatra	6.3	VII	1195	1798	2,501,798
17	12/01/2010	Haiti	Haiti	7.0	X	222,570	300,000	3,700,000
18	23/10/2011	Van (Turkey)	Eastern Turkey	7.1	VIII	604	4152	32,938
19	09/11/2011	Van (Turkey)	Eastern Turkey	5.6	VII	40	30	105
20	11/03/2011	Tōhoku (Japan)	Eastern Japan	9.1	IX	19,759	6242	2553
21	20/04/2013	Lushan (China)	Sichuan, Chongqing, Shaanxi	7.0	VIII	198	14,785	2,198,785
22	15/10/2013	Bohol (Philippines)	Philippines	7.2	IX	230	976	3,222,224
23	26/01/2014	Cephalonia Island (Greece)	Western Cephalonia	6.1	VII	0	0	-
24	03/02/2014	Cephalonia Island (Greece)	Western Cephalonia	5.9	VIII	0	10	-
25	25/04/2015	Gorkha (Nepal)	Kathmandu Valley, Everest	7.8	Χ	8831	17,932	5,639,722
26	16/04/2016	Ecuador	Ecuador, Colombia, Peru	7.8	VIII	672	6274	389,364
27	14/04/2016	Kumamoto (Japan)	Kumamoto Province, Kyushu, Japan	6.2	-	9	800	120,800
28	16/04/2016	Kumamoto (Japan)	Kumamoto Province, Kyushu, Japan	7.0	IX	49	1684	298,432

As shown in Table 1 and the distribution of earthquake epicenters in Figure 1, the earthquakes that have formed ideal conditions for the occurrence of infectious diseases in the post-disaster period have a magnitude equal to or greater than 5.6, with most of them distributed among major earthquakes (13 events in the magnitude class 7.0–7.9) and strong earthquakes (10 events in the magnitude class 6.0–6.9). The smallest contribution is made by moderate earthquakes (2 events in the magnitude class 5.0–5.9) and great earthquakes (3 events in the magnitude class greater than 8.0). The low contribution of moderate earthquakes is attributed to their limited impact on elements of the built environment (buildings and infrastructure) and thus to their low potential to cause adverse conditions for infectious diseases. The low contribution of the great earthquakes is attributed to the fact that these events occur less frequently worldwide.

In terms of their distribution, the earthquakes that have the potential to induce infectious diseases in the post-disaster period occur within major active fault systems worldwide, such as the Tethyan Alpine system (12 earthquakes in Italy, Greece, Turkey, Iran, Pakistan and China), the Circum Pacific Belt, better known as the Ring of Fire (15 earthquakes in Japan, Taiwan, Philippines, Indonesia, the Solomon Islands, the eastern part of North America, Costa Rica, Colombia, El Salvador, and Ecuador), and the Caribbean-Central America fault system (an earthquake in Haiti).

Reporting of the infectious disease occurrence after destructive earthquakes generated around the world between 1980 and 2016 showed that the incidence of infectious diseases increased after each of these earthquake events. Respiratory, gastrointestinal, and skin infections are the most common infections detected in the post-earthquake period. The presence and recording of wound infections is also pronounced. The timing and magnitude of an earthquake, its area of occurrence (proximity to active faults, coastlines and mountain fronts), the earthquake-triggered environmental effects, including landslides, hydrological anomalies, and tsunamis, and the synergy with different types of natural hazards can play an essential role in the increased incidence of infectious diseases.

Taking into account the information presented in Table 1, it is noted that the earthquakes that have triggered the emergence of sporadic cases, outbreaks, and epidemics of infectious diseases in the post-earthquake period are events of magnitude equal or greater than Mw = 5.6 with widespread and severe effects on the population, including thousands of human casualties, injured people, and homeless in need of immediate care and shelter. More specifically, more than 5 reports were found for the 2008 Wenchuan (China) Mw = 7.9 earthquake (*n* = 13), the 2015 Gorkha (Nepal) Mw = 7.8 earthquake (*n* = 11), the 1999 Izmit (Turkey) Mw = 7.6 earthquake (*n* = 9), the 1999 Chi-Chi (Taiwan) Mw = 7.7 earthquake (*n* = 9), the 2003 Bam (Iran) earthquake Mw = 6.6 (*n* = 8), the 2010 Haiti (*n* = 8) Mw = 7.0 earthquake, and the 2004 Indonesia Mw = 9.2 earthquake (*n* = 7) (Table 1).

## 4. Earthquake-Triggered Respiratory Infectious Diseases

**Figure 2:**
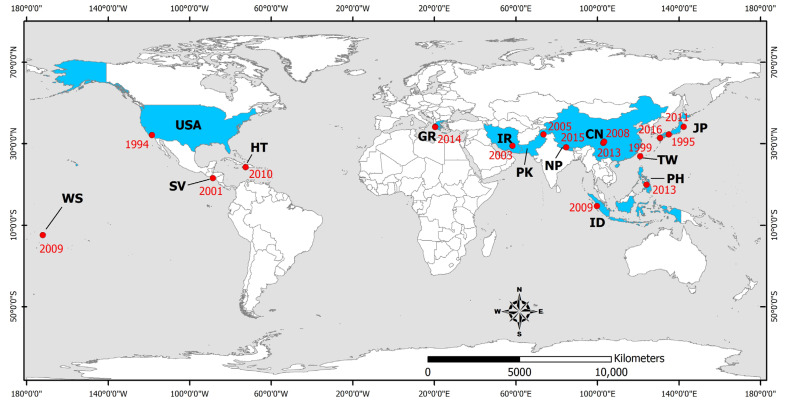
Distribution of countries affected by earthquakes that triggered the occurrence of respiratory tract infections. WS: Samoa, USA: United States, SV: El Salvador, HT: Haiti, GR: Greece, IR: Iran, PK: Pakistan, NP: Nepal, CN: China, JP: Japan, TW: Taiwan, PH: Philippines, ID: Indonesia. The epicenters of the studied earthquakes are also illustrated (red dots) along with the occurrence year (red numbers).

Upper respiratory tract infections were observed quite frequently in the short-term period after disasters induced by earthquakes generated in several continents, including North America (the 1994 Northridge [68,69], 2001 El Salvador [70,71], and 2010 Haiti earthquakes [72,73,74,75,76,77], Europe (the 2009 L’Aquila earthquake [78] and 2014 Cephalonia Island earthquakes [79]), and Asia (the 1995 Kobe [80], 1999 Chi-Chi [81,82], 2003 Bam [83,84], 2004 Sumatra-Andaman [85], 2005 Kashmir [86,87], 2009 Sumatra [88], 2009 Samoa [85], 2011 Tōhoku [85,89], 2013 Lushan [90,91], 2013 Bohol [92,93], 2015 Gorkha [94,95,96,97,98,99] and 2016 Kumamoto earthquakes [100]) (Figure 2). Most of the earthquake-affected people lived in overcrowded evacuation shelters, with inadequate air ventilation, unsafe drinking water, and poor personal hygiene being among the possible predisposing factors of contracting respiratory infectious diseases [70,71,72,80,81,82,83,84,87,88,90,92,95,96,98,100]. More details on the respiratory infectious diseases transmitted during the post-earthquake period in earthquake-affected areas are presented in brief in Table 2 and in detail below.

### 4.1. Viral-Associated Diseases

In addition to the number of injuries that increased due to the 1995 Kobe earthquake, the number of respiratory diseases, mainly pneumonia, increased about 4.5 times in one month [80]. In February 1995, 24 pneumonia patients with an average age of 79 years were admitted and the mortality rate was 25%. In contrast, one year earlier, the mortality rate was 14%, as only one of the seven pneumonia patients with an average age of 66.5 years died [80].

The number of acute respiratory infection cases detected in the affected area after the 1999 Chi-Chi (Taiwan) earthquake was higher than that of neighboring unaffected counties [81,82]. Most of the disaster victims lived in emergency camps [82]. It is noteworthy that the incidence of these infections decreased to normal expected levels four weeks after the earthquake [81] revealing the association between the earthquake occurrence and the emergence and transmission of infectious diseases among the affected people.

Surveillance of infections in the rural town of San Sebastian after the 2001 El Salvador earthquake showed that upper respiratory infections (30%) were the second most prevalent after skin infections [70].

Survivors of the 2003 Bam earthquake in Iran were temporarily housed in tents and received daily visits as part of an infectious disease surveillance system. Upper respiratory tract infections were recognized as the most common problem. Overall, 792 cases occurred 3 weeks after the earthquake due to the low temperatures, especially at night [83]. The study by Jafari et al. [84] confirmed that the most common cause of admission to health care centers was acute respiratory infection. Considering the total population of Bam after the earthquake (90,928 residents), the incidence of respiratory infection within 1 month was 686 per 10,000 inhabitants, corresponding to 6.86% of the total population [84].

A search of the medical records of all outpatients examined between August 2006 and December 2008, following the 2005 Kashmir earthquake in Pakistan, showed that the most common condition was viral upper respiratory tract infection (23%) [87].

After the 2009 Sumatra earthquake, 1015 patients were examined at two primary health care clinics. Respiratory diseases accounted for the most frequent diagnoses [88].

After the 2010 Haiti earthquake, approximately 42,361 cases were recorded during the period from 25 January to 24 April 2010. Nationally, the most commonly reported cases were acute respiratory infections (16.3%) [72].

Ten days after the 2013 Lushan earthquake in Sichuan Province (China), common infectious diseases recorded in children included respiratory infections, among others [90].

In the Philippines in 2013, disasters were attributed to different types of natural hazards: a flood, an earthquake, and a typhoon. Communicable infectious diseases were the predominant group of diseases recorded in all three types of disasters and included acute respiratory infections [92].

Cephalonia Island (Ionian Sea) is located in one of the most seismic active areas of Europe. It was affected by the early 2014 earthquakes, on January 26 and February 3 with Mw = 6.1 and Mw = 5.9, respectively. The earthquakes were generated during the winter period characterized by low temperatures and rainfall for several days between the two events, leading to an increase of respiratory infection cases [79].

Contagious airborne diseases were among the most common findings observed after the 2015 Gorkha earthquake [95]. Among the 108 pediatric patients examined, respiratory tract infections were observed in 42.3% of the patients [96]. The study conducted by Giri et al. [98] confirmed the results of Wang et al. [96]—that earthquakes can affect children in any age group and children are one of the most vulnerable population groups [98]. Among the 1057 patients examined, the percentage of patients requiring admission for pneumonia was significantly higher among children from areas and families significantly affected by the earthquake [98]. Therefore, ensuring well-functioning water and sanitation systems, temporary shelter and housing assistance, functional primary health care services, and effective systems for surveillance and registration of infectious diseases are vital for the livelihoods of displaced populations [95].

The two earthquakes that struck Kumamoto (Japan) in April 2016 caused evacuation in the earthquake-affected area. Evacuees were forced to spend an extended period of time in temporary camps and experienced upper respiratory tract infections, among other gastrointestinal and skin infections [100].

As regards the impact of earthquake-induced tsunami on public health, it is demonstrated that the harsh conditions following the Indian Ocean tsunami caused by the Mw = 9.2, Sumatra–Andaman earthquake on 26 December 2004, the Samoa tsunami caused by the Mw = 8.1, Samoa earthquake on September 29, 2009, and the Great East Japan tsunami caused by the Mw = 9.0, Tohoku (Japan) earthquake on 11 March 2011, have favored the emergence and incidence increase of respiratory infectious diseases [85]. Among tsunami survivors who had narrowly avoided drowning, polymicrobial respiratory infections (RIs) were frequently found. Throughout the period of influenza transmission, influenza outbreaks were frequently detected. Increased incidence of acute RI, measles transmission, and tuberculosis detection were all impacted by overcrowding in evacuation facilities [85]. More details on the respiratory infections following earthquake-induced tsunami can be found in the review conducted by Mavrouli et al. [85].

### 4.2. Fungal-Associated Diseases

#### Coccidioidomycosis

After the Northridge earthquake, between 24 January and 15 March 1994, Ventura County (California) experienced a major epidemic of coccidioidomycosis, a respiratory disease caused by inhalation of airborne spores of the dimorphic fungus *Coccidiodes immitis*, which grows in the upper layers of soil in limited semi-arid areas of the western hemisphere (e.g., southwestern United States, Mexico, and parts of Central and South America). Approximately 60% of infected individuals are asymptomatic. The disease most commonly presents as an influenza-like respiratory illness, although a wide range of clinical symptoms may occur. Overall, only 1 in 200 people infected with *C. immitis* develop diffuse diseases [68].

Landslides that occurred after the earthquake and its strong aftershocks in the Santa Susana Mountains located north of Simi Valley resulted in dust clouds that were dispersed into nearby valleys by northeasterly winds [69]. Following the landslides, the number of coccidioidomycosis cases in the region increased dramatically and peaked 2 weeks after the earthquake as 203 cases of coccidioidomycosis or valley fever were identified, of which 56% occurred in the town of Simi Valley.

Individuals who reported physical presence in dust clouds were 3 times more likely to be diagnosed with acute coccidioidomycosis than those who did not. The risk of being diagnosed with acute coccidioidomycosis increased with increasing duration of exposure and staying in dust clouds [68].

Both the location and timing of the outbreaks strongly suggest that the outbreak of coccidioidomycosis in Ventura County was caused when arthrospores of *C. immitis* spread with dust clouds due to landslides caused by the 1994 Northridge earthquake [69].

### 4.3. Mycobacterial-Associated Diseases

#### Tuberculosis

Following the 2013 Bohol (Philippines) earthquake, an assessment of the risk of *Mycobacterium tuberculosis* infection in children from earthquake-affected areas showed that of the 5476 children tested, 355 were positive for the tuberculin skin test, used to diagnose latent tuberculosis, and 16 had active tuberculosis. Although the prevalence of tuberculosis did not differ significantly between areas that were severely or less severely affected by the earthquake, living in a shelter with >25 people was found to have a significant effect on the tuberculosis incidence. Tuberculin skin test positivity appeared to be associated with older age, previous tuberculosis treatment, known contact with a person with tuberculosis, and living on a geographically isolated island. These data should be taken seriously in the design of national tuberculosis control programs, particularly with regard to the role that children’s vulnerability and geographically isolated communities play in the transmission and maintenance of tuberculosis [93].

In 2010, Haiti suffered a devastating earthquake that destroyed the healthcare infrastructure in the capital Port-au-Prince and left 1.5 million people homeless. Subsequently, there was an increase in reported tuberculosis in the affected population [75]. Even before the earthquake, Haiti had the highest incidence of tuberculosis in the Americas. About half of the tuberculosis cases occur in the Port-au-Prince area. The number of reported tuberculosis cases in Haiti increased after the earthquake, which may be due to an increase in the incidence of tuberculosis and/or improved methods of detecting cases [75].

Compared to previous national estimates (230 per 100,000 population), undiagnosed tuberculosis was three times higher in a shelter with internal displaced people (693 per 100,000) and five times higher in an urban slum (1165 per 100,000) [75]. Early detection of rising tuberculosis rates is a challenge, and data collection should be integrated into realistic disease response programs [75]. From January to June 2013, active tuberculosis was detected among residents of a slum in Port-au-Prince. Of the approximately 7500 residents screened, 394 (5%) had a cough lasting ≥2 weeks and 100 (25%) were diagnosed with active tuberculosis. In total, 144 tuberculosis cases were identified in 6 months (1920/100,000—national estimate 200/100,000) and a high rate of undiagnosed tuberculosis was found in Port-au-Prince even 3 years after the earthquake [76].

Six months after the 2008 Sichuan earthquake, tuberculosis cases recorded in hospitals in the affected Wenchuan region have increased compared to the same period of the previous year. Overall, 88.27% of patients lived in simply constructed temporary shelters in the affected area, among which only 58.06% met adequate sanitary conditions. Poor living conditions, overcrowding, excessive fatigue and stress, treatment interruption, and temporary inability to manage patients resulting from the disaster likely contributed to the worsening of tuberculosis epidemiology [101].

A 14-year-old girl, who was living with her family in a temporary emergency shelter, presented with fever, abdominal pain, and vomiting at a temporary health center in the earthquake-affected city of Bhakatpur in Gorkha (Nepal). Clinical abdominal examination indicated acute peritonitis. On laparotomy, three ileal perforations were identified, and histopathological examination showed cystic granulomas. Combined with the fact that her father had pulmonary tuberculosis with positive sputum, the child was diagnosed with tuberculous peritonitis and responded well to the administered anti-tuberculosis chemotherapy [99].

## 5. Earthquake-Triggered Gastrointestinal Diseases (Water- and Food-Borne Diseases)

**Figure 3:**
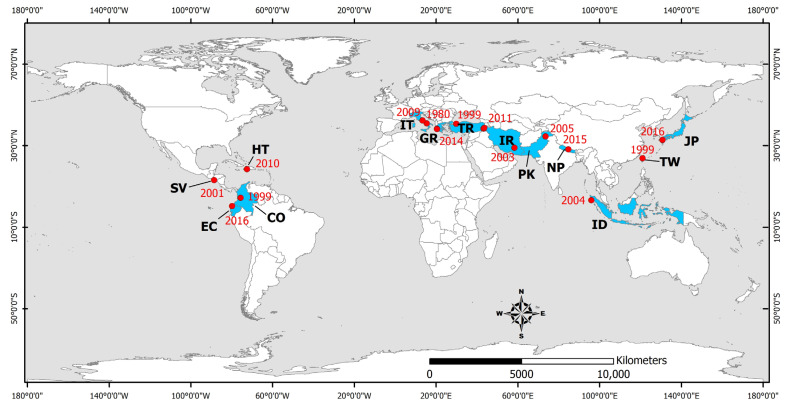
Countries affected by earthquake-triggered gastrointestinal infections. HT: Haiti, SV: El Salvador, EC: Ecuador, CO: Colombia, IT: Italy, GR: Greece, TR: Turkey, IR: Iran, PK: Pakistan, NP: Nepal, JP: Japan, TW: Taiwan, ID: Indonesia. The epicenters of the studied earthquakes are also illustrated (red dots) along with the occurrence year (red numbers).

Waterborne and foodborne diseases are mainly caused by the ingestion of water or food contaminated with pathogenic microorganisms (bacteria, viruses, and parasites) derived from human or animal feces. Gastrointestinal infections associated with earthquakes have been reported following the 1980 Irpinia (Italy) [102], 1999 Izmit (Turkey) [103,104,105,106,107,108], 1999 Chi-Chi (Taiwan) [81,82], 1999 Armenia (Colombia) [109], 2001 El Salvador [70,71], 2003 Bam (Iran) [83,84], 2004 Indian Ocean (Indonesia) [110], 2005 Kashmir (Pakistan) [87,111], 2009 L’Aquila (Italy) [112], 2010 Haiti [77,113], 2011 Van (Turkey) [114], 2014 Cephalonia earthquakes [79], 2015 Gorkha (Nepal) [94,98,115,116], and 2016 Kumamoto (Japan) earthquakes [100] (Figure 3). More details on the water- and food-borne infectious diseases transmitted during the post-earthquake period in earthquake-affected areas are presented in brief in Table 3 and in detail below.

Over a period of seven weeks after the 1980 Irpinia earthquake (Italy) 32 suspected outbreaks were reported, of which only two were confirmed: (a) an outbreak of gastroenteritis in a group of firefighters (39 cases) and (b) an outbreak of viral hepatitis (Hb negative) (6 cases) in a community in the province of Potenza adjacent to the earthquake zone [102].

After the 1999 Izmit earthquake, several factors, such as very hot weather conditions, close contact with the local population left homeless after the event, and the absence of proper water and sanitation systems in the area, could have increased the risk of infectious diseases among members of the Israeli Defense Force (IDF) search and rescue (SAR) team. A mild gastroenteritis outbreak was recorded among SAR personnel at the secondary rescue site in Chinargik town, where 30 out of 62 IDF soldiers complained of two to three loose or watery stools accompanied by abdominal pain. The incident was attributed to poor food handling practices because the cooked meats had been stored without refrigeration for several hours [103]. The low incidence of infectious diseases in the main rescue site in Golguk city can be attributed to the strict application of personal and environmental hygiene rules in the IDF camp, food handling procedures in the area where the teams worked, and immunization. In the secondary rescue area, where these measures were applied less strictly, the gastroenteritis infection rate was 48% [103].

Except from the respiratory infections, the 2014 Cephalonia Island (Greece) earthquakes also resulted in a gastrointestinal outbreak comprising 22 gastroenteritis cases among soldiers that developed diarrhea and abdominal pain 6–7 h after consuming a meal prepared by the same catering company. However, no clinical sample was collected for laboratory testing [79].

Members of search and rescue (SAR) teams and first responders visiting and operating in affected areas are characterized as vulnerable groups of travelers. Following the earthquakes in Nepal in April and May 2015, there was an influx of such teams in the affected areas. Common problems recorded among volunteers included traveler’s diarrhea and skin problems. It was found that the volunteers were not adequately prepared for the situations they were likely to face, suggesting that proper information and advice on health issues before acting to the affected area can help reduce the incidence of health problems among this particular group [97]. An assessment of morbidity among Israeli rescue team personnel in Nepal after the earthquake revealed that gastrointestinal complaints were by far the most common and significant morbidity (accompanied by diarrhea 44%, vomiting 22%, and fever 10%), followed by respiratory problems (16%) [94].

### 5.1. Bacterial-Associated Diseases

#### 5.1.1. Shigella

The distribution of shigellosis is worldwide and is estimated to cause around 600,000 deaths per year. Two thirds of cases and the majority of deaths are in children under 10 years of age. Outbreaks are observed in overcrowding conditions and where personal hygiene rules are not followed such as in prisons [117], day care centers [118], psychiatric clinics [119,120], and refugee camps [121,122]. A common mode of Shigella transmission is the consumption of food or water contaminated with fecal matter.

Immediately after the devastating 1999 Izmit (Turkey) earthquake, an infectious disease surveillance system was set up in Kocaeli province, which was the most affected by the earthquake. Waterborne microorganisms that were observed before the earthquake, such as *Shigella* species, *Salmonella* species, and *Giardia intestinalis*, could be the possible causes of diarrheal disease outbreaks due to the hot summer, damaged infrastructure, and difficulties in obtaining safe drinking water [104]. Diarrheal diseases increased gradually after 20 August 1999 and decreased to the normally expected level on 15 September 1999. Of the identified causes, *Shigella* species were the most common isolates [104].

Following the 1999 Chi-Chi (Taiwan) earthquake, 15 cases of shigellosis were recorded in Nantou County from 21 September to 31 October 1999. Four and eleven cases were reported in Nantou County during the corresponding periods in 1997 and 1998, respectively [81]. The increased incidence of diarrheal diseases could be attributed to seasonal variations and overcrowding conditions [81].

#### 5.1.2. Salmonella Enterica

An epidemic of *Salmonella enterica* infection occurred in children in the town of L’Aquila (Abruzzo region, central Italy) between June 2013 and October 2014, four years after the 6 April 2009 devastating earthquake [112]. Salmonella infection occurred in 155 children, aged 1 to 15 years, of which 44 were hospitalized due to severe dehydration, electrolyte imbalances, and persisting fever, despite the administration of antipyretic and antibiotic drugs. The high proportion of hospitalized children highlights the emergence of a highly pathogenic *S. enterica* strain probably following contamination of natural springs’ water due to geological processes that occurred during the devastating earthquake [112].

#### 5.1.3. Tularemia

Wild and domestic animals, such as rabbits or rodents, as well as ticks, can transmit tularemia to humans. There are four modes of transmission, including ingestion of contaminated food or water, handling infected animals, insect bites, and inhalation of contaminated dust [123]. In Europe, ingestion of contaminated water from streams, lakes, ponds, and rivers is the main mode of *Francisella tularensis* transmission [124].

The first case of tularemia in the town of Golcuk, Kocaeli province was recorded after 5 patients were admitted to a central hospital in the province in January 2005 [107]. They all came from a new settlement built after the 1999 earthquake at high altitude near a forest area. The main mode of transmission was ingestion of contaminated water from natural springs that had been safely used for decades before the devastating 1999 earthquake [107].

#### 5.1.4. Cholera

Cholera is an acute diarrheal disease caused by the intestine being attacked by the enterotoxin produced by the bacterium *Vibrio cholerae*. Transmission of the disease occurs through the consumption of contaminated food or water [125]. Water is usually contaminated by patient feces, which in turn can directly or indirectly contaminate food. Food can also be contaminated by dirty hands during meal preparation or when consumed. In endemic areas, cholera is most commonly found in areas with inadequate water supply and poor sanitary conditions.

After the 2010 Haiti earthquake, cholera was thought to have been reintroduced to Haiti after the country had been cholera-free for almost a century [126]. On 19 October 2010, the Haitian Ministry of Public Health and Population was informed of a sudden increase in patients with acute watery diarrhea and dehydration in the Artibonite and Plateau Centrale regions. Laboratory tests confirmed *V. cholerae*, Ogawa biotype, serogroup O1, as the causative pathogenic agent. Several of the patients reported drinking untreated water from the Artibonite River and not using toilets [113]. Therefore, the disease occurrence was due to inability to access safe water, poor sanitation, and overcrowding conditions. Given its highly infectious nature and lack of pre-existing immunity, the cholera epidemic following the 2010 earthquake had a significant impact on the health of Haitian children, with the CDC reporting over 82,000 cases of cholera in children under five years of age. The collapse of public health and healthcare infrastructure, as well as limited financial resources due to the earthquake, made it even more difficult to effectively respond to the cholera epidemic [77].

In addition to Haiti, cholera outbreaks occurred after the 2015 Gorkha earthquake in Kathmandu, the capital and largest city of Nepal. Twenty-nine people developed cholera from *V. cholerae* serogroup O1 serotype Ogawa due to drinking contaminated water in densely populated areas of Kuleshwor and Kalimati districts in Kathmandu [115]. According to Sekine et al. [116], 169 cases of acute watery diarrhea caused by *V. cholerae* were reported in 2015, of which 150 were identified in Kathmandu Valley.

#### 5.1.5. Helicobacter Pylori

One month after the 2011 Van earthquake in Turkey, 209 dyspeptic patients undergoing gastroscopy were screened for the presence of *H. pylori* infection [114]. A significantly higher prevalence of *H. pylori* was found in disaster survivors compared to patients screened during the same period before the earthquake. These results suggest that a seismic event could contribute to the development of *H. pylori* infection in individuals living in the disaster-affected areas [114].

### 5.2. Protozoa-Associated Diseases

#### Giardiasis

Giardiasis is an infection of the digestive tract by the protozoan *Giardia lamblia* that colonizes the small intestine of mammals. Humans become infected accidentally by ingesting protozoan cysts either through ingestion of feces-contaminated food and water or through direct contact with infected individuals [127].

On 25 January 1999, a strong earthquake destroyed 70% of the houses in the city of Armenia (Colombia). Emergency shelters were organized and operated for up to 2 years after the disaster. Parasitological studies carried out on the affected population showed that giardiasis was the most common parasitic infection [109]. Factors that were associated with *Giardia* infection included the use of communal toilets (versus individual toilets) and water supply from municipal sewers (versus water supply from individual tanks) [109]. A high prevalence of *Giardia lamblia* was found in children living in camps after the Armenian earthquake. Giardiasis is an emerging disease after destructive events and appropriate prevention measures should be implemented during these conditions [109].

The application of hygiene measures and adequate washing of fruits and vegetables before consumption help to avoid ingestion of contaminated cysts. Protozoan cysts are known to survive in chlorinated water but can be destroyed by boiling, drying, and freeze/thaw cycles. Filtration processes have been shown to be effective in removing *Giardia* cysts from drinking water at a rate of about 99%, but the use of more than one disinfectant is required [109].

Two earthquakes in the north-western region of Turkey destroyed 80% of the houses and schools in the city of Düzce in 1999. Oztürk et al. [105] compared 2 groups of children living and studying in different socioeconomic conditions as a result of the earthquake. They found that the rate of Giardiasis and Enterobiasis was significantly higher in children who were still living and studying in temporary settlements and schools even years after the earthquakes (*p* < 0.05) [105].

### 5.3. Virus-Associated Diseases

#### 5.3.1. Rotavirus

The 2005 Kashmir earthquake caused widespread contamination of drinking water sources and contributed to a rotavirus outbreak between October and December 2005. Rotavirus is the leading cause of severe diarrhea worldwide among children younger than 5 years and is responsible for around 40% of related deaths, estimated at over 500,000 per year [128]. In Kashmir, rotavirus transmitted by the fecal–oral route led to acute diarrheal disease among infants and young children [111]. The epidemic was brought under control after the establishment of clinics within emergency camps and awareness-raising actions about the need to drink boiled water and adhere to safer hygiene practices [111].

#### 5.3.2. Hepatitis A and E

Hepatitis A and E infections remain an important public health concern in many developing countries, where poor socioeconomic conditions and high population density contribute to the transmission of these viral infections [129]. However, hepatitis A can emerge even in urban areas if there is overcrowding, lack of clean water, or inadequate sewage disposal and treatment systems [130,131].

Sencan et al. [106] investigated the prevalence of intestinally transmitted hepatitis among children living in Golyaka and Düzce camps after the İzmit and Düzce earthquakes, respectively, which struck northwestern Turkey twice in less than 3 months. After the Izmit earthquake, irregularities were observed in the provision of drinking water and the maintenance of good sanitation conditions due to confusion. The second earthquake shook the city of Düzce, but the necessary measures were quickly implemented to organize camps for earthquake victims, provide drinking water and food, and distribute financial aid. The prevalence of HAV in children living in the temporary camps in Golyaka and Düzce was 68.8% and 44.4%, respectively, while the prevalence of HEV in children was 17.2% and 4.7%, respectively. The prevalence of HAV and HEV in children was lower than that in endemic areas but higher than that recorded in developed countries. Moreover, it was found that the prevalence of HAV and HEV was higher in Golyaka district compared to Düzce district, because in the latter case the state mechanism was alert and responded immediately by taking necessary measures, such as providing clean water and food, and preparing an emergency action plan to prevent these infectious diseases [106].

Kaya et al. [108] assessed the incidence rates of hepatitis A and E in children living in Duzce four years after the 1999 earthquakes, compared to rates determined immediately after the earthquakes [108]. It was found that hepatitis A was still common in the pediatric age groups, whereas hepatitis E occurred relatively rarely [108].

## 6. Earthquake-Triggered Vector-Borne Diseases

**Figure 4:**
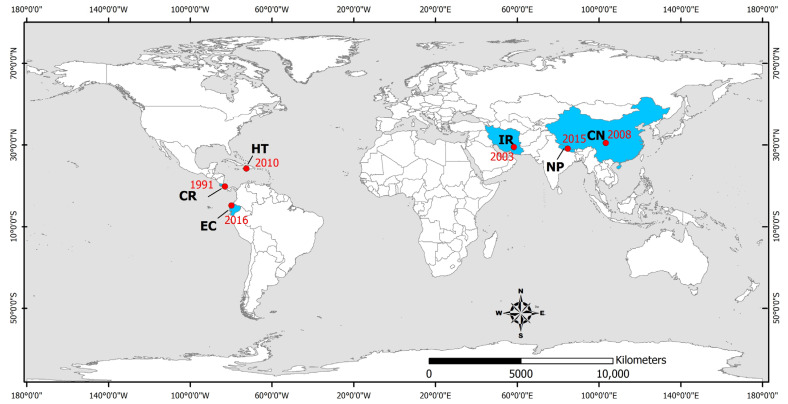
Countries affected by earthquake-triggered vector-borne infectious diseases. HT: Haiti, CR: Costa Rica, EC: Ecuador, IR: Iran, NP: Nepal, CN: China. The epicenters of the studied earthquakes are also illustrated (red dots), along with the occurrence year (red numbers).

Vector-borne diseases are infections transmitted by the bite of infected arthropod species, such as mosquitoes, midges, ticks, and mites. They were detected in areas affected by the 1991 Costa Rica [132], 2003 Bam (Iran) [133,134,135,136,137,138], 2008 Sichuan (China) [139], 2010 Haiti [73,74,77], 2015 Gorkha (Nepal) [140,141], and 2016 Ecuador [142,143,144,145] earthquakes (Figure 4). More details on the vector-borne infectious diseases transmitted during the post-earthquake period in earthquake-affected areas are presented in brief in Table 4 and in detail below.

### 6.1. Bacterial-Associated Diseases

#### Scrub Typhus

Scrub typhus is caused by *Orientia tsutsugamushi* and is transmitted to humans through bites of infected mites found in forests, bushland, gardens, and beaches. Most cases are recorded in rural areas of Southeast Asia, Indonesia, Japan, China, India, and northern Australia [146].

A few months after the 2015 Gorkha earthquake in Nepal and the subsequent strong aftershocks, scrub typhus outbreaks were reported in different parts of the country, particularly in earthquake-affected areas [140,141]. Overcrowding and unsanitary living conditions in temporary emergency shelters could have contributed to increased contact between vectors, pathogens, and humans. Building collapse led to increased circulation of rodents carrying mites infected with *O. tsutsugamushi* and consequently increased the likelihood of human exposure to these infected vectors. The detection of *O. tsutsugamushi* in humans, rodents, and mites in the affected areas, as well as the widespread reports of scrub typhus cases across the country for 3 consecutive years, confirms the ongoing transmission of *O. tsutsugamushi* with a firmly established ecology in Nepal [140]. The 2015 earthquake weakened the health system and caused problems in diagnosis, treatment, and surveillance of the disease, resulting in a major epidemic in 2016 [141]. From 2015 to 2017, 1239 scrub typhus cases were confirmed, with the largest epidemic occurring in 2016 with 831 cases. Therefore, it was considered necessary to strengthen the health care system of the country to enhance systematic surveillance, achieve early detection and reporting of cases, and implement immediate measures for the prevention and treatment of the disease [141].

### 6.2. Protozoan-Associated Diseases

#### 6.2.1. Malaria

In 1991, a huge increase in malaria cases was recorded following the earthquake and floods in Costa Rica because of an increase in the mosquito population caused by deforestation and changes in river flow patterns [132]. Sáenz et al. [132] epidemiologically investigated the incidence of malaria in two time periods: (a) 22 months before the Limón earthquake in April 1991 and (b) 13 months after this catastrophic event. It was found that in some areas there was an increase in malaria incidence of 1600% and 4700% above the average monthly rate for the period before the earthquake. This was associated with (a) changes in human behavior, such as increased exposure to mosquitoes when sleeping outdoors and the temporary cessation of malaria control activities, (b) changes in the environment that favored mosquito breeding, such as deforestation from landslides, destruction of river dams, and (c) the August 1991 floods [132].

Malaria caused by *Plasmodium falciparum* is endemic in Haiti and the main mosquito vector is *Anopheles albimanus*, which is mainly active at dawn and dusk and prefers to live and feed outdoors. Consequently, populations living outdoors or in temporary shelters, as well as thousands of first responders in Haiti, are at significant risk of developing malaria from *P. falciparum*. During the period 12 January to 25 February 2020, CDC received reports of 11 laboratory-confirmed cases of *P. falciparum* malaria occurring in Haiti (7 U.S. residents—immediate emergency responders, 3 Haitian residents, and 1 U.S. resident/traveler) [73].

#### 6.2.2. Leishmaniasis

Cutaneous leishmaniasis is a disease with a wide range of clinical manifestations and occurs mainly in seven countries: Afghanistan, Algeria, Algeria, Brazil, Iran, Iran, Peru, Saudi Arabia, and Syria [147]. Two forms of cutaneous leishmaniasis occur in the Bam region, anthropogenic and zoonotic, caused by *Leishmania tropica* and *L. major*, respectively [133].

Detecting and reporting of cutaneous leishmaniasis cases in the rural town of Zarindasht in the southern Iranian province of Fars from April 2002 to April 2004 showed that 2003 earthquakes may have led to an outbreak of the disease as the annual incidence increased from 58.6 confirmed cases/100,000 in the 12 months before the earthquakes to 864 confirmed cases/100,000 (at the peak of the outbreak) in the following 12 months [133]. Most of the skin lesions observed in Zarindasht were mainly on the face [133].

Construction of new settlements, expansion of villages, development of previously uninhabited areas, and poor management of waste and debris piles are risk factors that have contributed to the increase of the disease incidence [147]. The 2003 Bam earthquake caused the production of 10 million tons of debris, creating suitable and favorable conditions for the proliferation of sandflies, the main vector of *Leishmania* species. Since 2004, the epidemiology of cutaneous leishmaniasis has changed with the emergence of new outbreaks and non-healing clinical forms that persist for a long time [135,148].

All age groups were affected, especially children aged 10 years, which probably suggests the existence of (a) acquired immunity in older city dwellers and (b) behavioral patterns of children, such as outdoor play, that increase their exposure to sandflies [149].

It is noteworthy that although both earthquakes accounted for only one human loss, they caused huge ecological changes, disrupted the normal activities of residents, and forced many of them to sleep outdoors until the threat of aftershocks and further damage to buildings was reduced. The ecological changes that created an ideal environment for the development of the sandflies population and the aftershock behavior of the city residents (debris removal, housing rehabilitation, degraded living conditions) contributed to the increased exposure of the affected population to the sandflies bites [149].

Despite considerable efforts and implementation of various approaches to reduce transmission of the disease, cases of cutaneous leishmaniasis increased to epidemic proportions after the earthquake, particularly during 2006–2008 in Bam city [134,136]. During the last 20 years (1993–2012), 20,999 cases of cutaneous leishmaniasis occurred, including 6731 before (1993–2003) and 14,268 after the Bam earthquake (2003–2012) [138].

It is worth noting that, although the prevalence of cutaneous leishmaniasis cases has increased after the earthquake [134], the severity of cases has decreased significantly, which is mainly attributed to the establishment of a health clinic specialized in cases of cutaneous leishmaniasis [137].

Most cases of cutaneous leishmaniasis occurred in unimmunized men. In the post-earthquake period, the Bam population increased significantly, mainly due to the massive movement of workers and contractors undertaking large-scale construction projects. After the earthquake, men aged >20 years were mainly infected, the majority of them with skin lesions located on the extremities (hands or feet) [134,138]. Job opportunities and the development of new projects in Bam led to a non-immunized workforce in areas where cutaneous leishmaniasis is endemic, resulting in an increase of cutaneous leishmaniasis incidence in the earthquake-affected area [134].

The Sichuan earthquake in China occurred in May 2008, when the hot and humid weather was beneficial for insect breeding and multiplication. Zhang et al. [139] examined the incidence of insect-borne diseases such as Japanese encephalitis, visceral leishmaniasis (Kala-azar), and malaria before and after the earthquake in Longnan city. There was no significant difference in the incidence of Japanese encephalitis after the earthquake compared to 2005 and 2007. Regarding cutaneous leishmaniasis, there was no significant difference in its incidence in 2008, 2009, 2010, and 2011 compared to 2007, but the number of cases in 2008, 2009, and 2010 was slightly higher than the other years. However, by 2011, this number had decreased to pre-earthquake levels [139].

### 6.3. Viral-Associated Diseases

#### Zika Virus

Zika virus (ZIKV) is an arbovirus transmitted to humans by the mosquitoes *Aedes aegypti* and *Aedes albopictus*, as well as by other routes of transmission, including sexual transmission [150]. Despite the relatively mild clinical course in symptomatic healthy adults, the effects on fetal development can be devastating [142].

The 2016 Ecuador earthquake contributed to the increase of reported Zika virus infection cases [145]. Building collapse forced people to remain outdoors, exposed them to a chaotic urban space full of debris and industrial waste (plastic bottles), prevented waste collection, and left them without safe drinking water. In addition, access to medical care was significantly delayed due to the earthquake. Most people were on long waiting lists or had to travel to neighboring provinces to seek medical care [145].

As of 16 April 2016, a total of 92 cases of ZIKV had been recorded across the country. In the aftermath of the earthquake, this number rose rapidly to 1106 total cases in just 3 months. In total, 80% of these cases were reported in Manabi province, the area most affected by the earthquake [144]. Reina Ortiz et al. [143] found that the significant increase in the number of ZIKV cases was particularly evident in areas that were severely affected and suffered significant loss of life and property from the earthquake compared to those that were mildly affected.

The ZIKV outbreak in the city of Manabi, Ecuador, after the 2016 earthquake, demonstrates the negative impact of natural hazards in socially vulnerable areas where climatic conditions are not considered normal for the region. An extremely strong El Niño event created environmental conditions that favored the proliferation of mosquito vectors just as ZIKV first appeared in Ecuador [142].

The increase in ZIKV incidence may be associated with the movement of population groups and overcrowding that may increase exposure to virus vectors. Destruction of property and sewage and water management systems may increase the number of Aedes aegypti breeding sites. Stressful conditions alter the immune status and increase the susceptibility of the population to developing symptomatic disease [144].

## 7. Wound and Skin Infections

Wound and skin infection refers to the infection of tissues by one or more types of microorganisms. The type of wound infection depends mainly on the environment in which the injury took place, the extent of the injury, the microorganisms present on the skin of the injured person, the microorganisms to which the person has been exposed during wound healing, and the general health and immune status of individuals.

Traumatic injuries that occur after an earthquake disrupt the balance of the immune system and increase the predisposition to infectious complications. Being trapped under rubble for a long period of time can cause severe crush injuries, leading to an increased risk of exposure to pathogens.

**Figure 5:**
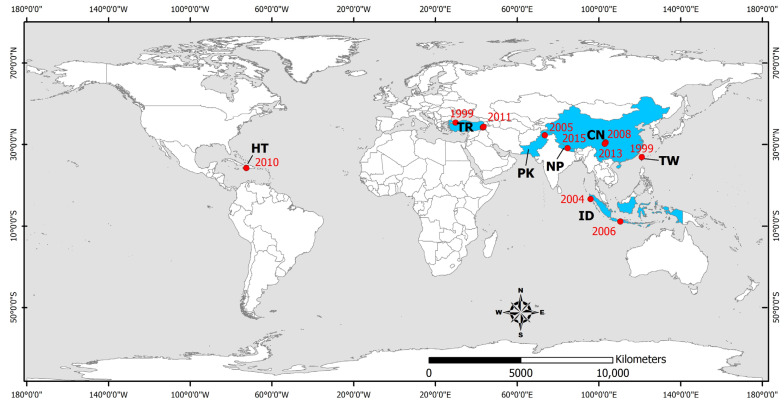
Countries affected by earthquake-triggered wound and skin infections. HT: Haiti, TR: Turkey, PK: Pakistan, NP: Nepal, CN: China, ID: Indonesia. The epicenters of the studied earthquakes are also illustrated (red dots), along with the occurrence year (red numbers).

Wound and skin infections were reported after the 1999 Izmit (Turkey) [151,152,153], the 1999 Chi-Chi [154], the 2005 Kashmir (Pakistan) [86,155], the 2004 Sumatra-Andaman (Indonesia) [156,157,158,159,160,161,162], the 2006 Yokyakarta (Indonesia) [162,163], the 2008 Sichuan (China) [164,165,166,167,168,169,170,171,172,173,174], the 2010 Haiti [77,175,176], the 2011 Van (Turkey) [177], the 2013 Lushan [91], and the 2015 Gorkha (Nepal) [178] earthquakes (Figure 5, Table 5).

Most patients in the 2008 Sichuan earthquake were buried under rubble with soil, bricks, or stone. Time under debris and time from injury to treatment was associated with the wound infection occurrence. Victims who survived the earthquake were often severely injured. The incidence of wound infections was high due to inadequate provision of medical personnel, surgical equipment and antibiotics, subsequent rainfall, and high temperature in earthquake-affected areas [167]. The most common pathogen isolated from wound samples was *Staphylococcus aureus*, but only 24.4% of all isolated microorganisms were Gram-positive bacteria and 73.2% were Gram-negative, such as *Escherichia coli*, *Acinetobacter baumannii*, *Enterobacter cloacae*, and *Pseudomonas aeruginosa* [166,167,168,169].

A similar picture was seen in the studies by Kang et al. [165] and Zhang et al. [173] where Gram-negative bacilli isolation from infected wound samples was predominant. Within 1 month of the Sichuan earthquake, 50 injured children were diagnosed with wound infections. The most frequently isolated pathogens were *A. baumannii* (27%), *E. cloacae* (18%), and *P. aeruginosa* (13%). However, this distribution of pathogens differed from that in hospitalized cases in the year before the earthquake. The pathogens most frequently isolated in the pre-earthquake period were *E. coli* (27%), *S. aureus* (23%), and coagulase-negative staphylococci (9%) [167]. Meanwhile, the isolation rate of multidrug-resistant bacteria was higher during the post-disaster period [167].

After the 2010 Haiti earthquake, the majority of earthquake victims were admitted to hospitals for limb injuries requiring orthopedic surgery and treatment of infected wounds. Overall, 77% of the wound infections were polymicrobial and involved predominantly Gram-negative bacteria, which were resistant to the antimicrobials recommended in current CDC and WHO guidelines [175].

After the 2005 Kashmir earthquake in Pakistan, Gram-negative bacteria (*P. aeruginosa*, *Enterobacter* spp., and *Acinetobacter* spp.) were the most common microorganisms isolated from polymicrobial wound infections [155]. The predominance of Gram-negative bacteria in wound infections was also confirmed after the 2015 Gorkha earthquake that hit Nepal [178].

Recording these changes in the spectrum and resistance of pathogens isolated after earthquakes can contribute to the timely administration of effective treatment after similar seismic events like the 2008 Sichuan (China) earthquake [167]. Earthquake-affected injured people should be given broad-spectrum antibiotics such as cephalosporins and macrolides. The selection of appropriate antibiotics can greatly enhance the effectiveness of early specific treatments and prevent serious complications of wounds in future natural hazards [164].

Crush syndrome can lead to prolonged hospitalization and is associated with an increased risk of subsequent infection [168]. A high rate of isolation of multidrug-resistant bacteria and hospital-acquired infections were observed after the 2008 Sichuan earthquake due to severe injury, long hospitalization as a result of acute renal failure and immune system dysfunction [168,170]. Sepsis and wound infection were more common in patients who underwent fasciotomy or amputation than in those who did not undergo such procedures after the 1999 Chi-Chi (Taiwan) and the 2008 Sichuan (China) earthquakes [154,170,174]. Vascular catheters, urinary catheters, and long-term stays in intensive care units (ICUs) may also increase susceptibility to hospital-acquired infections, as happened after the 1999 Izmit [152] and the 2011 Van earthquakes [177] in Turkey. Oncül et al. [153] highlighted that *Acinetobacter* was the main bacterial isolate identified from patients with wound infections attributed to the 1999 Izmit earthquake compared to the almost non-existent prevalence of *Acinetobacter* infections in the ICU of the same hospital. *S. aureus* and *E. coli* were the most common pathogens at the initial stage of hospitalization. With a prolonged ICU stay, *A. baumannii* and *Klebsiella pneumoniae* gradually became the dominant pathogens [91].

It is noteworthy that the aforementioned isolated bacteria differed significantly from the isolation pattern recorded in the 2004 Indian Ocean earthquake and tsunami, where the most commonly reported infectious agents were *Aeromonas* spp., *E. coli*, *K. pneumoniae*, *P. aeruginosa*, and *Proteus* spp. [156,158,159].

The high frequency of Gram-negative bacilli isolation from wound cultures is probably explained by the mode of wounding. Gram-negative bacilli are naturally present in soil and water and people are probably exposed to these bacteria at the time of injury. It is also likely that these infections are hospital-acquired because most patients come from front-line hospitals, where drug resistance rates can be high [167,173].

Laboratory microbiological analysis is essential for the optimal selection of antibiotics to treat infection, as well as for preventing and possibly reducing the risk of hospital-acquired infection. In addition, effective management of infection contributes to better wound care and therefore leads to lower mortality and disability rates [173].

Craniocerebral injuries are rarely vulnerable to infection due to the abundant blood supply. However, among individuals injured in the Sichuan earthquake, scalp wound infections were recorded quite frequently [172]. Gram-positive bacteria were the most frequent microorganisms isolated (64.4%), including *S. aureus* and *S. epidermidis*. Gram-negative bacteria (35.6%), including *E. cloacae*, *K. pneumoniae*, and *Serratia rubidaea*, were detected in a smaller number of samples in contrast to the pattern seen in wound infections in other body areas [172].

Certain measures can improve the prognosis of patients with severe infection, including early recognition of infection, early identification of microorganisms, rational use of antimicrobial agents with the guidance of infectious disease experts, targeted therapy to boost the immune system, necessary surgical removal of infected foci, early debridement, and strict surveillance for hospital-acquired infections [91].

### 7.1. Tetanus

Tetanus is an acute infectious disease caused by the exotoxin of *Clostridium tetani*. The bacterial spores are mainly found in soil, human feces, and on the surface of rusty objects [179].

Despite being easily preventable with a highly effective vaccine, tetanus remains a major source of morbidity and mortality worldwide. The death rate from tetanus remains high in developing countries affected by natural hazards, where tetanus vaccination coverage is often low or non-existent. Successful treatment of tetanus depends on prompt diagnosis, timely administration of treatment, administration of sedative and muscle relaxant drugs, maintaining an open airway, and mechanical respiratory support in the management of respiratory failure [180]. Tetanus cases have been reported after the 2004 Indonesia earthquake, the 2005 Kashmir earthquake, the 2006 Yogyakarta earthquake, and the 2010 Haiti earthquake [86,161,163,176].

In January 2005, an epidemic of tetanus was detected among the tsunami survivors in Indonesia. Of the 106 cases, 79% were over 25 years old (the median age was 40 years) and 62% were male [160]. The case fatality ratio was 18.9%, higher among older patients [160]. Fifteen patients presented with severe tetanus associated with surface trauma, three of whom had a history of submersion. Supplies to treat tetanus cases in this outbreak were initially limited, as disaster relief agencies were not prepared for the resulting tetanus outbreak [161]. In addition to the 106 cases that occurred in Acheh district, another 71 cases were recorded in Yogyakarta. In both outbreaks, most patients were injured during evacuation or post-disaster rehabilitation. Difficult access to health care due to limited transport or hospital facilities, as well as low vaccination coverage and lack of awareness about the risk of tetanus, contributed to delayed treatment. Prevention of post-disaster tetanus outbreaks can be achieved by increasing vaccination coverage, raising community awareness of the risk of tetanus, improving the treatment administered for wound infections, and establishing a surveillance system for wound-related infectious diseases [162].

The tetanus cases detected following the 2004 Indian Ocean earthquake and tsunami was the largest cluster reported after a natural disaster or an event with huge number of casualties, surpassed only by the 2005 Kashmir earthquake after which 139 cases were recorded [160]. The enormous number of injuries sustained during the disaster and the poor prior immunization status of the population are reflected in both tetanus outbreaks. In Islamabad, Pakistan, a total of 51 multi-injured patients developed tetanus requiring respiratory support, and 22 of these died because of the severe nature of the disease and ineffective treatment [86].

Following the 2006 Yogyakarta earthquake, 26 tetanus cases were recorded, including 8 fatalities. Significant predictors of death included the distance from the patient’s residence to the hospital and the type of hospital [163].

### 7.2. Gas Gangrene (Myonecrosis)

Gas gangrene or myonecrosis is a highly fatal deep soft tissue infection caused by *Clostridium perfringens*. A prerequisite for the occurrence of gas gangrene is wound contamination in such a way that anaerobic conditions are created, i.e., either the wound is deep or the tissues from the injury have been necrosed. Gas gangrene cases were detected in victims of the Sichuan earthquake and several of these were confirmed after culture to have resulted from *Clostridium perfringens* infection [169,171].

### 7.3. Other Skin Infections

In the first three months after the 1999 Izmit earthquake, registrations to the outpatient clinic of a dermatology department included infections/infestations and dermatoses mainly due to damaged infrastructures and unhygienic life conditions. In the following 3 months, erythematous squamous, pruritus, neurocutaneous dermatoses, and eczemas predominated and were due to psychoemotional factors related to the earthquake [151].

The evaluation of skin problems recorded at a general hospital in Banda Aceh after the 2004 Indian Ocean earthquake showed that the most common skin problems were infections/infestations, followed by eczemas and traumatic skin disorders, which were more common in men [157]. The prevalence of infections/infestations, traumatic skin disorders, and contact dermatitis probably increased due to exposure to a hazardous environment, unsanitary living conditions, and contact with various objects both during and after the tsunami [157].

## 8. Risk Factors and Preventive Measures

Earthquakes alter existing environmental conditions, resulting in direct public health impacts, including occurrence of sporadic cases, outbreaks, and epidemics of infectious diseases in the affected population and those involved in emergency response and recovery actions.

Strong ground motion alters the stability conditions on slopes, resulting in slides and rockfalls on natural and artificial slopes. The resulting dust clouds have a high potential to adversely affect public health, especially when carried by prevailing winds to residential rural and urban areas. A typical example of such an effect is the outbreak of coccidioidomycosis that occurred when airborne spores of *Coccidioides immitis* were transferred to Ventura County with dust clouds generated during the 1994 Northridge, California earthquake [68,69].

The vertical displacement of the seabed and offshore earthquake-triggered landslides can generate large tsunamis with significant impacts on infrastructure and the population of coastal areas. Typical examples are tsunamis generated in individual parts of the Ring of Fire, including the Indian Ocean tsunami on 26 December 2004, the Samoa Islands (South Pacific Ocean) tsunami on 29 September 2009, and the Japan tsunami on 11 March 2011, which have flattened extensive coastal areas with thousands of human casualties and widespread post-event public health impacts [85].

Apart from the above direct effects of secondary seismic phenomena on public health, there are also indirect effects, mainly resulting from damage and malfunction of the main elements of the built environment. The strong ground shaking and the resulting severe structural damage to residential buildings create an emergency need for immediate sheltering of thousands of people in places, whether organized or not, where overcrowding prevails and sanitary conditions deteriorate.

Damage to health care facilities causes problems and delays in providing first aid and immediate medical care to the affected people. The destruction of parts of the road network causes temporary disruption to transport services and difficulties in accessing essential supplies and emergency services immediately. These effects on lifelines are characterized by high potential to induce the occurrence of infectious diseases and even result in human casualties among earthquake survivors.

### 8.1. Risk Factors for Infectious Diseases Occurence

As concluded from the above literature review on earthquake-triggered infectious diseases, the risk factors that can lead to the occurrence of sporadic cases, outbreaks, and epidemics of infectious diseases in an earthquake-affected area are summarized as follows [85,163,181,182,183,184]:

- Heavy structural damage to critical healthcare infrastructure, including facilities and buildings, remained unfixed during the emergency response and the subsequent recovery, causing delayed management and treatment of severe infectious diseases cases.
- Lack of awareness and provision of early or real-time warning for the upcoming and the ongoing events, resulting in little time for preparedness or evacuation.
- Severely injured earthquake and tsunami survivors highly exposed to high pathogen densities in soil and water.
- Immense changes in climate and aggravating weather conditions in the emergency response phase including dramatic temperature changes.
- Prolonged physical exposure to and inhalation of:
  ○ airborne particulate matter due to large dust clouds formed by earthquake-triggered landslides.
  ○ airborne particulate matter due to contaminated ejecta dust formed by earthquake-triggered liquefaction phenomena with a destructive impact on water and sewage systems.
- Prolonged physical exposure to and aspiration of contaminated water due to tsunami generation and impact on the coastal residential zones.
- Emergency shelters and evacuation camps with:
  ○ High population density and overcrowding.
  ○ Poor living conditions comprising small places for individuals and families and insufficient equipment for the homeless and evacuees (blankets, bed clothes, sleeping bags, etc.).
  ○ Lack of clean running water due to damage and contamination of the water supply systems and water sources, respectively.
  ○ Unsanitary conditions (poor hand hygiene) due to a shortage of personal hygiene items.
  ○ Malnutrition due to insufficient food provision and shortage of long-lasting food supplies.
  ○ Insufficient equipment comprising essentials and poor heating, ventilation and air conditioning systems among others.
  ○ Rodent/vector infestation.
- Increased exposure to vectors and rodents due to earthquake-triggered ecological changes, leading to extensive formation of potential breeding and feeding sites of vectors and rodents.
- The weak immune system of vulnerable groups of the affected population comprising the elderly, the chronically ill individuals, and young children.
- The dependency of young children, people with disabilities, and elders of the affected communities seeking assistance with daily activities.
- Poor socio-economic conditions, including large percentage of low education level, a large part of the population living below the national poverty line leading to insufficient personal hygiene and denial of risk, disinterest, and ignorance of risk, as well as poorly constructed buildings and infrastructures.
- Poor health education and training on infectious disease prevention and control.
- Lack of emergency preparedness and training of residents and medical staff for infectious disease prevention.
- Insufficient or low vaccination coverage due to limited vaccination campaigns and short supplies.
- Close contact with cases and activities and interaction in areas where clusters of infectious disease cases have been observed.

### 8.2. Prevention Strategies and Actions for Mitigating the Risk of Earthquake-Triggered Infectious Diseases

The knowledge of factors underlying earthquake-triggered infectious diseases can contribute to the adoption and application of effective prevention strategies. These measures should be taken in the light of a more multihazard approach to environmental, disaster, and crisis management, related to different types of hazards. This need has begun to emerge in recent decades as the interactions between mainly natural hazards became increasingly evident [185,186]. It also emerged recently between hazards of different types (natural and biological), mainly due to the occurrence of earthquakes and extreme hydrometeorological events amid the COVID-19 pandemic [187,188].

#### 8.2.1. Establishment of a Proper Disease Surveillance System

One of the widely proposed measures for the prevention of infectious diseases after an earthquake and its effects is the establishment of a proper disease surveillance system. The main purpose of the surveillance system is initially to immediately and effectively identify the pre- and post-disaster infectious disease cases and trends through the collection, compilation, and analysis of information related to the number of cases of various diseases.

Through this rapid identification of infectious disease cases, it is possible to contribute effectively to improvement of disease trends monitoring, validity of early warnings, support for vaccine development, the development of more effective prevention strategies and thus to a more complete assessment of the public health burden of infectious diseases. Among the key structural elements of this surveillance system are the alert and response components.

One of the most important prerequisites for strengthening the early warning component of a surveillance system is the regular exchange of information between the competent authorities. Proper disease surveillance after a disaster is further enhanced by prompt diagnosis and subsequent treatment, which should be expanded to all high-risk parts of the earthquake-affected areas in order to prevent the acquisition of infectious diseases and the subsequent emergence of outbreaks and epidemics.

The significance of the infectious disease surveillance systems should be emphasized in emergency shelters for the implementation of infection prevention and control measures. The emergency shelters are critical post-disaster infrastructures, providing immediate temporary shelter to those who leave their homes due to severe structural damage or evacuation of an area affected by both the earthquake and its accompanying phenomena. However, in the first days or hours after the earthquake occurrence that has led to a surge of evacuees and internal displaced people and heavy structural damage of the healthcare facilities and life lines in affected areas, emergency shelters are the places where the first cases of infectious diseases may occur, leading to related outbreaks or even epidemics.

#### 8.2.2. Prevention of Infectious Diseases in Emergency Shelters

Typical examples of sporadic cases, outbreaks, and epidemics of infectious diseases come from Japan and the emergency shelters that were set up to accommodate those in need of immediate shelter and food after the 2011 earthquake and the subsequent tsunami that hit much of the coastal zone in the eastern part of the country [182,184]. Emergency shelters were so crowded that evacuees were forced to lay down on their backs on the floor without being able to turn over during sleep. Many of them even hesitated to cough to avoid disturbing others in the same area, while others ignored their oral hygiene. The above, combined with the synergy of frequent aspiration, malnutrition, and low temperatures, led to people contracting pneumonia, especially the elderly and infants [182,183].

In order to prevent such adverse effects during staying in emergency shelters, it is necessary to conduct actions to mainly cover the gaps in the organization and operation of these places. Thus, ample amounts of bottled water and canned and dry food should be available, adequate ventilation (heating and air-conditioning) should be provided, personal protective equipment (face mask, disposable gloves, disinfectants) should be distributed to prevent the spread of respiratory tract infections, mosquito nets and insect repellents should be distributed and spraying should be carried out to prevent vector-borne diseases, and appropriate pharmaceutical material and effective vaccines should be available in sufficient quantities. As regards the awareness-raising and information of evacuees and staff, material comprising posters and leaflets should be posted in several sites of the shelters for enhanced prevention of infectious diseases not only in the emergency shelters but also in the affected community.

In order to avoid overcrowding in emergency shelters, more shelters of the same or different type should be constructed. This approach was successfully used in the case of devastating earthquakes that hit Greece during the COVID-19 pandemic, where maintaining physical distance between evacuees was a measure to limit the spread of SARS-CoV-2 in the earthquake-affected communities [187,188,189]. In particular, after the 2020 Samos Mw = 7.0 earthquake during the second wave of the pandemic in Greece, hotels were used to house homeless and evacuees in order to avoid overcrowding in the open-air earthquake camps that had been set up in football fields on the island [190]. After the 2021 Thessaly earthquakes during the third pandemic wave in the country, more types of emergency shelters were used, including campervans, container-type structures, hotel rooms, and houses of relatives and close friends [188,189]. With this measure as part of a multi-hazard approach to disaster and crisis management of geophysical hazards in the midst of an evolving biological hazard, there was no burden on the evolution of the pandemic in earthquake-affected areas.

Infectious disease mitigation measures in emergency shelters can be divided into (i) measures for first responders and personnel involved in the emergency response, (ii) mitigation measures for accommodated staff and visitors in emergency shelters, (iii) administrative and engineering controls in emergency shelters, including changes in the layout of facilities and distribution practices of supplies, and (iv) designation of isolation facilities to separate suspected cases of infectious diseases [188].

#### 8.2.3. Prevention of Infectious Diseases among First Responders and Affected Population

When an earthquake causes extensive structural damage to the built environment and a major impact on the population of the affected area, search and rescue teams are mobilized not only from the services of the affected country and neighboring countries but also from other countries on other continents with different epidemiological characteristics. In order to avoid sporadic cases, outbreaks, or even epidemics of infectious diseases in non-endemic countries or the addition of epidemic risk factors to existing ones, all people involved and responding to disaster management comprising rescuers, volunteer teams, and aid organizations should respect the fundamental principle of all assistance, which is initially not to harm. With this in mind, measures should be taken to protect the health of both first responders and the affected population. With regard to the first aspect of protecting those involved in the immediate response, strict adherence to personal hygiene and safety and environmental sanitation protocols should be applied in areas where SAR operations are conducted and in emergency shelters. Furthermore, adherence to good hygiene practice in the handling, preparation, and distribution of food should also be applied in the areas where these teams operate [103,188,190]. A good practice was presented by Gdalevich and Ashkenazi [103] and was related to the experience of the Israel Defense Force (IDF) SAR team during the aftermath of the 1999 Izmit (Turkey) earthquake.

In the affected area where the SAR team was deployed, due to the absence of running water, abundant amounts of mineral water and baby wipes were used for washing. Food for the team was limited to prepackaged or canned field rations (canned meat, vegetables, jam, etc.). The use of local products in meal preparation and their consumption was prohibited. Close medical guidance was given to all food handlers and environmental hygiene supervision was continuously carried out during the mission. These methods, combined with immunization, kept morbidity to a minimum. In another region, where all of the above was not strictly followed, there was a 48% gastroenteritis infection rate [103].

As far as the protection of the local population is concerned, the same strict adherence should be applied to personal and environmental hygiene rules. The example of the cholera epidemic following the 2010 Haitian earthquake, which caused human losses greater than those attributed to the earthquake’s impact on the country’s buildings and infrastructure, is typical. Many researchers have addressed this epidemic [113,191,192,193,194,195,196]. The cholera outbreak in a country that was not endemic was attributed to a synergy of poor personal and environmental hygiene practices in relief staff facilities and poor adherence to personal hygiene rules by most of the local population. In order to reduce such phenomena, the necessary actions should be as follows:

- Ensuring the safety of drinking water and sewerage systems not only in either temporary or permanent relief camps but also in areas characterized by high risk
- Compliance with the existing safety and hygiene measures
- Where existing measures are not sufficient, educational activities for hygiene and sanitation awareness raising should be conducted, aiming for the integration of safe water and hand hygiene best practices into households, communities, and countries
- Large-scale preventive vaccination and medicine programs for several infectious diseases should be implemented, along with the establishment of a proper disease surveillance program and a long–term empowering and strengthening of the public-sector health system.

In order to provide relief efforts to a region systematically, data and information on the prevalent endemic and epidemic infectious diseases in the disaster-affected area should be collected shortly after the earthquake occurrence. A quick evaluation of the existing infectious disease patterns should be also assessed, along with the demographic conditions of the affected area, including the number, size, and location of residential areas and population density, the availability of safe clean running water and adequate personal and environmental sanitation facilities, as well as the nutrition status and the immunization coverage of the disaster-affected population [194].

#### 8.2.4. Measures for Mitigating the Risk of Respiratory Infectious Diseases

With regard to respiratory infections caused by secondary earthquake environmental effects, mainly including landslides and liquefaction, measures deal with limiting the exposure of affected people to dust clouds generated during landslides and the clearance of areas covered from extensive liquefied deposits [69,197,198]. Immediate post-earthquake measures should include guidance to the general public to avoid dust clouds or areas of heavy dust. Preventive actions include awareness-raising among clinicians, earth scientists, field workers, and the public containing information about the risks posed by airborne infectious diseases and their impact on public health. Furthermore, ways to minimize exposure to infection and recognize possible symptoms should be introduced to all involved in risk mitigation actions, including earthworks for removing landslide material and liquefied deposits from covered parts of the road network.

Increased incidence of respiratory infections could be attributed to seasonal variations and overcrowding conditions in emergency shelters in the earthquake-affected areas. The correlation between shelter crowding and an increased ARI incidence rate was demonstrated by Kawano et al. [199] following the Great Eastern Japan earthquake and tsunami. The minimum amount of space needed at shelters to prevent ARI outbreaks in the evacuation shelters was found to be 5.5 m^2^ per person. The UN Refugee Agency (UNHCR) [200] developed guidelines for the minimum living space at shelters, proposing a minimum 4.5–5.5 m^2^ per person in cold climates and 3.5 m^2^ per person in tropical, warm climates. These recommendations could facilitate emergency evacuation planning and result in the reduction of ARI incidence at emergency shelters.

#### 8.2.5. Measures for Mitigating the Risk of Waterborne Diseases

To avoid the occurrence of waterborne diseases during the emergency in earthquake-affected areas, one of the most effective proposed measures is the examination of water and sewage systems for detecting non-structural and structural damage with potential to affect public health. It is a practice that has been applied in all developed countries for decades. If non-structural and structural damage is found in these systems, chlorination or even an initial supply from a different source is applied and then disinfection follows [201,202]. Individual toilets should be used instead of shared ones, and camp areas need to have trench latrines available to prevent open-air defecation. Provision for hand-washing with soap and water is required. The protection of water sources should be strengthened, and hygiene and sanitation should be ensured.

#### 8.2.6. Measures for Mitigating the Risk of Vector- and Rodent-Borne Diseases

To reduce the risk of vector- and rodent-borne diseases throughout the earthquake-affected areas, public health authorities should give priority to surveillance. The implementation of response activities and control measures, as well as the improvement of preparedness efforts aimed at emerging vector- and rodent-borne diseases, depends on the immediate recognition and identification of local vector and rodent species, environmental factors, and breeding habitats that influence local disease transmission.

Information on how to avoid contact with water, especially if skin abrasion happens, avoid touching eyes, nose, or mouth, identify rodent- and vector-borne infection symptoms, and seek immediate medical help and advice if you become ill should be distributed to the general public and disaster management officials. Since misdiagnosis or delayed diagnosis has serious clinical implications, early detection of signs and symptoms, appropriate treatment, and application of disease-specific therapies are essential to reduce morbidity and mortality. The earthquake-affected population should also stay away from places like uncontrolled waste disposal sites, garbage dumps, and areas with stagnant water where they might come into contact with contaminated surface water or infected animal urine.

A rapid risk assessment should be performed within the first week of the earthquake’s occurrence by gathering data on the earthquake-affected area and population, with a focus on internally displaced people, as well as the risk of infectious disease outbreaks and disruptions to the public health infrastructure. The establishment of adequate disease surveillance systems and the identification of suitable interventions for managing and mitigating the negative effects of infectious disease outbreaks that happen concurrently with or after an earthquake disaster are both made possible by recorded data.

#### 8.2.7. Education, Training, and Awareness-Raising Actions

The improvement of health surveillance systems, the maintenance and provision of effective and efficient health care services, and the reduction of associated mortality and morbidity may all be aided by education, training, and awareness-raising initiatives for the identification and management of infectious diseases.

It may be possible to prevent the onset of an infectious disease outbreak during the post-disaster phase or to mitigate and eliminate the negative effects on the public health after the onset of an infectious disease outbreak by taking awareness-raising and informational actions in areas with high seismic risk and susceptibility to seismic effects. In addition to making it simple to implement personal protective measures to prevent infection, these actions allow for effective management of an impending outbreak.

Flexible preparedness planning and measures that can quickly and effectively incorporate unforeseen circumstances and associated negative effects are required in terms of long-term prevention. This strategy necessitates expanding our understanding of the connection between infectious diseases and disasters induced by natural hazards. Planning for emergency response to disasters by natural hazards should include measures for preventing infectious disease outbreaks and mitigating their effects on the affected population and the staff involved in emergency response and recovery.

The public, in particular residents belonging to particular groups within communities who may be more susceptible than others to the negative effects of earthquakes and may therefore suffer the most from the generation of such events and subsequent infectious disease outbreaks, should also be informed of these measures in a timely and effective manner, in addition to the staff involved in the prevention and management of these emergencies. The elderly, people with disabilities, children, women, people with low incomes, the homeless, and migrants are some of these groups.

## 9. Conclusions

Earthquakes occur in specific zones where active faults have been formed. A rupture along these active faults results in primary and secondary effects with considerable impact on the built environment and the local population of the affected areas.

The synergy of pre-existing conditions and changes caused by the earthquake and its primary and secondary effects, combined with the physiographic characteristics of the affected area and the socio-economic characteristics of the affected communities, have high potential to cause public health impacts, including sporadic cases, outbreaks, and epidemics of infectious diseases, which may lead to human losses, resulting in a delay of the recovery process in the earthquake-affected area.

This study shows that earthquake-triggered infectious diseases have occurred not only in areas devastated by destructive earthquakes with hundreds to thousands of human casualties, thousands to tens of thousands injured, and tens of thousands to millions internally displaced people, but also in areas affected by earthquakes with an extensive impact on elements of the built environment (buildings and lifelines) but with no direct impact on human life, including losses and injuries.

The risk factors that favor the incidence of infectious diseases in earthquake-affected areas are related to the earthquake magnitude and intensity, the extent of its primary and secondary effects, the demographic and epidemiological characteristics of the affected area, and the level of education and living conditions of the affected communities, as well as the damage to public health structures and facilities.

The infectious diseases that have been recorded to date following the occurrence of earthquakes around the world are classified into (a) respiratory infections, (b) gastrointestinal diseases (water- and food-borne diseases), (c) vector-borne diseases, and (d) wound and skin infections.

Regarding measures for the prevention and management of earthquake-triggered infectious diseases, the most effective and widely recommended measure by almost all relevant published research is the establishment of an appropriate disease surveillance system. Such a system should be able to effectively identify infectious disease trends before and after the earthquake and contribute to a full assessment of the public health burden of the infectious diseases and their management. This system should be governed by a multiparametric, interdisciplinary, and multihazard approach. This approach is considered imperative, especially in recent years, where the parallel occurrence and evolution of geophysical and biological hazards and related disasters with public health implications is more frequent than ever before.

## Figures and Tables

**Table 2:** Respiratory infectious diseases transmitted during the post-earthquake period in earthquake-affected areas.

Earthquake Occurrence (DD/MM/YYYY)	Earthquake Affected Area	Infectious Diseases (Causative Factors–Cases, Outbreaks, Epidemics)	References
17/01/1994	Northridge California, United States	Outbreak of coccidioidomycosis (*Coccidiodes immitis*), 203 cases (including 3 deaths)	[68]
		Outbreak of coccidioidomycosis (*C. immitis*)	[69]
17/01/1995	Kobe, Japan	Increase in the number of patients with respiratory diseases by 4.5 times	[80]
21/09/1999	Chi-Chi, Taiwan	Acute respiratory infections	[81]
		Upper respiratory tract infection	[82]
13/01/2001	El Salvador	Upper respiratory infections (30%)	[70]
		Respiratory infections	[71]
26/12/2003	Bam, Iran	Respiratory infections (6.86% of the total population within 1 month)	[84]
		Respiratory tract infections	[83]
08/10/2005	Kashmir, Pakistan	Viral upper respiratory tract infection (23%)	[87]
12/05/2008	Sichuan, China	Increase of tuberculosis cases in hospitals of the affected area	[101]
30/09/2009	Sumatra, Indonesia	Respiratory infections	[88]
12/01/2010	Haiti	Acute respiratory infection (16.3%)	[72]
		Increase in tuberculosis in the affected population: 3-fold in a camp for internally displaced persons (693/100,000) and 5-fold in an urban slum (1165/100,000)	[75]
		Tuberculosis	[76]
11/03/2011	Tōhoku, Japan	43% of cases-community pneumonia (*Streptococcus pneumoniae*, *Moraxella catarrhalis* and *Haemophilus influenzae*)	[89]
20/04/2013	Lushan, China	Respiratory tract infections	[90]
		Respiratory infection (45.7%)	[91]
15/10/2013	Bohol, Philippines	Acute respiratory infections	[92]
		476/3555 children: positive to tuberculin skin reaction–TST, 16 with active tuberculosis	[93]
26/01/2014 03/02/2014	Cephalonia, Greece	Increase of respiratory infection cases	[79]
25/04/2015	Gorkha, Nepal	Pneumonia and post-streptococcal glomerulonephritis: high incidence among children from affected areas	[98]
		Upper respiratory tract infections	[95]
		Infections of the respiratory tract (42.3%)	[96]
		1 case of tuberculous peritonitis (1 girl 14 years old with fever, abdominal pain and vomiting)	[99]
14/04/2016, 16/04/2016	Kumamoto, Japan	Upper respiratory infections	[100]

**Table 3:** Water- and food-borne infectious diseases transmitted during the post-earthquake period in earthquake-affected areas.

Earthquake Occurrence (DD/MM/YYYY)	Earthquake Affected Area	Infectious Diseases (Causative Factors–Cases, Outbreaks, Epidemics)	References
23/11/1980	Irpinia, Italy	1 outbreak of gastroenteritis (39 cases, firefighters) 1 outbreak of viral hepatitis (6 cases, city of Potenza)	[102]
17/08/1999	Izmit, Turkey	*Giardia lamblia* and *Enterobius vermicularis* infections in children still living and studying in temporary settlements and schools even years after the earthquakes	[105]
		Hepatitis A and E	[108]
		Hepatitis A and E	[106]
		Tularemia outbreak (*Francisella tularensis*, 5 cases)	[107]
		A mild gastroenteritis outbreak among SAR personnel (two to three loose or watery stools accompanied by abdominal pain)	[103]
		Current increase in diarrheal infections (Shigella)	[104]
21/09/1999	Chi-Chi, Taiwan	Acute gastroenteritis (15 shigellosis cases)	[81]
		Acute gastroenteritis	[82]
25/01/1999	Armenia, Colombia	Giardiasis (*Giardia lamblia*)	[109]
13/01/2001	El Salvador	Gastrointestinal infections	[71]
26/12/2003	Bam, Iran	Gastrointestinal infections (0.81% of the total population within 1 month)	[84]
		Diarrheal diseases	[83]
26/12/2004	Indonesia	Tsunami survivors: waterborne infections (85% of children under 5 years old: diarrhea, 100% of the population had no access to clean drinking water and sanitation systems).	[110]
08/10/2005	Kashmir, Pakistan	Rotavirus outbreak	[111]
		Acute digestive disease (14.3%)	[87]
06/04/2009	L’Aquila, Italy	*Salmonella enterica* epidemic in children	[112]
12/01/2010	Haiti	Cholera and cholera-like disease	[77]
		Cholera outbreak	[113]
23/10/2011 09/11/2011	Van, Turkey	Significantly higher prevalence of *Helicobacter pylori* in dyspeptic patients-disaster survivors compared to dyspeptic patients in the pre-disaster period	[114]
26/01/2014 03/02/2014	Cephalonia, Greece	Gastroenteritis outbreak (22 gastroenteritis cases among soldiers)	[79]
25/04/2015	Gorkha, Nepal	Gastrointestinal infections	[94,97]
		Acute gastroenteritis: high incidence among children from affected areas	[98]
		Cholera (*Vibrio cholerae* serogroup 01 Ogawa serotype)	[115]
		Acute watery diarrhea (*Vibrio cholerae*): 169 cases, of which 150 were in the Kathmandu Valley	[116]
14/04/2016, 16/04/2016	Kumamoto, Japan	Gastrointestinal infections	[100]

**Table 4:** Vector-borne infectious diseases transmitted during the post-earthquake period in earthquake-affected areas.

Εarthquake Occurrence (DD/MM/YYYY)	Earthquake Affected Area	Infectious Diseases (Causative Factors–Cases, Outbreaks, Epidemics)	References
22/04/1991	Limon, Costa Rica	Malaria (*Plasmodium falciparum*)	[132]
26/12/2003	Bam, Iran	Cutaneous leishmaniasis (20,999 cases (1993–2012): 6731 before and 14,268 after the earthquake	[138]
		Anthroponic cutaneous leishmaniasis: increase in annual incidence from 58.6 cases/100,000 in the 12 months before the earthquakes to 864 cases/100,000 in the following 12 months	[133]
		Cutaneous leishmaniasis	[134,135,136,137]
12/05/2008	Sichuan, China	Visceral leishmaniasis	[139]
12/01/2010	Haiti	Suspected malaria (10.3%)	[72]
		11 laboratory-confirmed cases of *P. falciparum* malaria (7 US residents-emergency responders, 2 Haitians, 1 US traveler)	[73]
		Malaria	[77]
		*P. falciparum* malaria (76/255 patients)	[74]
25/04/2015	Gorkha, Nepal	Outbreak of scrub typhus (*Orientia tsutsugamushi*)	[140,141]
16/04/2016	Ecuador	Zika virus outbreak	[142,144,145]
		Zika virus outbreak (89 cases in the pre-earthquake period-2103 in the post-earthquake period)	[143]

**Table 5:** Wound and skin infectious diseases transmitted during the post-earthquake period in earthquake-affected areas.

Εarthquake Occurrence (DD/MM/YYYY)	Earthquake-Affected Area	Infectious Diseases (Causative Factors/Cases, Outbreaks, Epidemics)	References
17/08/1999	Izmit, Turkey	Infections/infestations, cutaneous superficial fungal infections (Tinea pedis), cases of viral skin diseases, insect bites	[151]
		Ιnfectious complications (wound infections): gram-negative bacteria (mainly *Acinetobacter* spp.), *Staphylococcus* spp.	[152]
		Wound infections: Gram-negative bacteria (*Acinetobacter baumanii*, *P. aeruginosa*, *Escherichia coli*, *Klebsiella pneumoniae*, *Stenotrophomonas maltophilia*) and *Staphylococcus* spp. (630 injured)	[153]
21/09/1999	Chi-Chi, Taiwan	Wound infections—Crush syndrome	[154]
26/12/2004	Indonesia	Wound infections: tetanus (106 cases), *Clostridium tetani*	[162]
		Ιnfections—infestations, traumatic skin lesions, and contact dermatitis	[157]
		Wound infections (*Aeromonas* spp., *E. coli*, *K. pneumoniae*, *P. aeruginosa*, and *Proteus* spp.)	[156]
		Skin and soft tissue infections	[158]
		Ιnfected superficial wounds on the limbs and face (recurrence: necrosis of underlying tissues, need for repeated cleaning, and dressing of wounds)	[159]
		Tetanus outbreak	[160,161]
08/10/2005	Kashmir, Pakistan	Gas gangrene of the limbs and tetanus requiring respiratory support (51 patients with tetanus, of whom 22 died)	[86]
		Wound infections: *P. aeruginosa*, *Enterobacter* spp. and *Acinetobacter* spp. (multi-resistant strains)	[155]
27/05/2006	Yogyakarta, Indonesia	Wound infections: tetanus (71 cases)	[162]
		Wound infections: tetanus (26 cases)	[163]
12/05/2008	Sichuan, China	Wound infections (*E. coli*, *S. aureus*, *S. haemolyticus*, *A. baumanii*, *A. cloacae*, *P. aeruginosa*, C-type chain coccus, and *Bacillus aerogenes capsulatus*), gas gangrene (*Clostridium perfringens*)	[164]
		67 probable cases (2.41%) of gas gangrene of which 5 were confirmed by culture (*C. perfringens*)	[171]
		Crush syndrome–wound infections: *A. baumanii*, *P. aeruginosa*, *E. cloacae*, and *E.coli*	[170]
		Wound infections: Gram-negative bacilli, Gram-positive bacteria, *Candida* spp., Gram-negative cocci, *Clostridium sordelli*	[165]
		Skull wound infections: Gram-positive bacteria (*S. aureus*, *S. epidermidis*), Gram-negative bacteria (*E. cloacae*, *K. pneumoniae*, *Serratia rubidaea*)	[172]
		Wound infections in 50 children: Gram-positive bacteria (16%), Gram-negative bacteria (82%) (*A. baumannii*, *E. cloacae*, *P. aeruginosa*), 1 month after the earthquake	[167]
		Wound infections (24.4% Gram-positive bacteria: *Staphylococcus aureus*, −73.2% Gram-negative bacteria: *Escherichia coli*, *Acinetobacter baumannii*, *Enterobacter cloacae*, and *P. aeruginosa*)	[166]
		Crush syndrome—wound infections: *A. baumannii*, *E. coli*, *S. aureus*, gas gangrene	[169]
		Wound infections: Gram-negative bacteria	[168]
		Wound infections: *A. baumannii*, *Burkholderia cepacia*, *S. aureus*, and *Enterococcus* spp.	[173]
		Crush syndrome	[174]
12/01/2010	Haiti	Wound/skin infections	[77]
		Wound infections (polymicrobial, 89% Gram-negative bacteria, antimicrobial resistant)	[175]
		Wound infections: tetanus (2 cases)	[176]
23/10/2011 09/11/2011	Van, Turkey	Wound infections: Gram-negative aerobic bacteria and *A. baumannii*, *P. aeruginosa*, *E. coli*, and *E. faecium*	[177]
20/04/2013	Lushan, China	Intracranial infection (initial stage of hospitalization: *S. aureus* and *E. coli*—prolonged stay in ICU: *A. baumannii* and *K. pneumoniae*	[91]
25/04/2015	Gorkha, Nepal	56 human losses: 68% Gram-negative bacilli (55%, Enterobacteriaceae)	[178]

## Data Availability

Not applicable.
